# Diagnostic Errors Induced by a Wrong a Priori Diagnosis: A Prospective Randomized Simulator-Based Trial

**DOI:** 10.3390/jcm10040826

**Published:** 2021-02-18

**Authors:** Felix M.L. Meyer, Mark G. Filipovic, Gianmarco M. Balestra, Kai Tisljar, Timur Sellmann, Stephan Marsch

**Affiliations:** 1 Department of Intensive Care, Kantonsspital Luzern, 6000 Luzern, Switzerland; felix.meyer@luks.ch; 2 Institute of Anesthesiology, Kantonsspital Winterthur, 8400 Winterthur, Switzerland; mark.filipovic@ksw.ch; 3 Department of Intensive Care, University of Basel Hospital, 4031 Basel, Switzerland; gianmarco.balestra@usb.ch (G.M.B.); kai.tisljar@usb.ch (K.T.); 4 Department of Anaesthesiology, Witten/Herdecke University, 58455 Witten, Germany; t.sellmann@bethesda.de; 5 Department of Anaesthesiology, Bethesda Hospital, 47053 Duisburg, Germany

**Keywords:** diagnostic error, randomized controlled trial, myocardial infarction, pulmonary embolism, simulation

## Abstract

Preventive strategies against diagnostic errors require the knowledge of underlying mechanisms. We examined the effects of a wrong a priori diagnosis on diagnostic accuracy of a focussed assessment in an acute myocardial infarction scenario. One-hundred-and-fifty-six medical students (cohort 1) were randomized to three study arms differing in the a priori diagnosis revealed: no diagnosis (control group), myocardial infarction (correct diagnosis group), and pulmonary embolism (wrong diagnosis group). Forty-four physicians (cohort 2) were randomized to the control group and the wrong diagnosis group. Primary endpoint was the participants’ final presumptive diagnosis. Among students, the correct diagnosis of an acute myocardial infarction was made by 48/52 (92%) in the control group, 49/52 (94%) in the correct diagnosis group, and 14/52 (27%) in the wrong diagnosis group (*p* < 0.001 vs. both other groups). Among physicians, the correct diagnosis was made by 20/21 (95%) in the control group and 15/23 (65%) in the wrong diagnosis group (*p* = 0.023). In the wrong diagnosis group, 31/52 (60%) students and 6/23 (19%) physicians indicated their initially given wrong a priori diagnosis pulmonary embolism as final diagnosis. A wrong a priori diagnosis significantly increases the likelihood of a diagnostic error during a subsequent patient encounter.

## 1. Introduction/Background

Compared to a vast variety of medical errors, diagnostic errors have only recently gained political and academic attention [1,2,3,4]. The 2015 report “Improving diagnosis in health care” of the Institute of Medicine defines a diagnostic error as failure to establish an accurate and timely explanation of the patient’s health problem or communicating that explanation to the patient [1]. Because of their central role in medical decision making and strong influence on subsequent procedures and therapy, faults in diagnosis can trigger far-reaching consequences [5,6]. Among malpractice claims, diagnostic errors appear to be the most common, most costly, and most dangerous of medical mistakes [7]. The true incidence of diagnostic errors is difficult to establish. Based on the currently available data, a fair estimate of the prevalence of diagnostic errors is approximatively 5% and most people will likely be affected by at least one diagnostic error during their lifetime [1,8,9,10].

The current knowledge on diagnostic errors mainly originates from retrospective error analysis and observational studies, both prone to hindsight bias [11]. In addition, not all errors can be detected retrospectively. Indeed, available data from prospective studies suggest that the incidence of diagnostic errors and their consequences like inappropriate or even dangerous management may be significantly higher than estimated from observational studies [6,12,13,14,15].

Patients with acute and severe illness presenting at emergency departments are generally triaged during a first patient–provider encounter. The rapid evolution of their disease and the limited opportunities to detect and correct errors in the settings of acute care makes these patients particularly vulnerable to harm related to a delayed or wrong diagnosis [16]. Thus, the diagnostic accuracy in the first patient–provider encounter is paramount for patients’ safety. Indeed, the implementation of systematic cross-checking between emergency physicians was associated with a significant reduction in adverse events [17].

Patients presenting to emergency departments often hold a suspected diagnosis of a pre-treating physician. Suspected diagnoses may be reported by a variety of sources like other doctors, paramedics, triage nurses, or from the patients themselves, and may be correct or wrong. To the best of our knowledge, there are no data on the influence of an a priori diagnosis on potential diagnostic errors during a subsequent patient encounter.

Accordingly, the aim of our study was to test the hypothesis that a wrong a priori diagnosis of a referring physician will increase the likelihood of wrong final diagnoses.

## 2. Methods

### 2.1. Study Design

This is an investigator-initiated prospective randomized controlled single-blind trial, the participants not being aware of the purpose of the study. The trial (ClinicalTrials.gov NCT04659265) was approved by the regional ethical committee (EKNZ 85/04), and all participants gave written informed consent.

### 2.2. Participants

Two cohorts were studied: In cohort 1, medical students in their 4th year at the University of Basel were offered voluntary workshops at our institution. The present study was conducted as part of these workshops. Second, the results of the first cohort prompted us to perform a study with physicians (residents and fellows in internal medicine and intensive care medicine) participating in simulator workshops at our institution (cohort 2).

At the beginning of the workshop, participants were asked to fill in a questionnaire. The questionnaire for the medical students included (a) the Big Five personality traits (neuroticism, extraversion, openness, agreeableness, conscientiousness) [18]; (b) Rosenberg’s self-esteem scale [19]; (c) the task to diagnose the cardiac rhythm in 12 one-lead ECG rhythms strips (each correct diagnosis was counted as one point resulting in a maximum of 12 attainable points); (d) the task to list the typical symptoms of an acute myocardial infarction in bullet point form (each of the following items were counted as one point resulting in a maximum of six attainable points: chest pain; radiation of the pain; pain described as constricting, compressing, or squeezing; at least one accompanied vegetative symptom; dyspnoea; and fear); and (e) the task to list the typical signs symptoms of four further medical emergencies (anaphylactic shock, trauma management, cardiac arrest, and syncope) in bullet point form (answers were coded using a predefined checklist). The questionnaire for the physicians included the Big Five personality traits and Rosenberg’s self-esteem scale.

### 2.3. Simulator and Scenario

The study was conducted using a patient simulator. All participants received a standardised instruction of the patient simulator (human patient simulator, SimMan^®^, Laerdal Medical AS, Stavanger, Norway). This simulator’s features include among others, palpable pulses, spontaneous breathing with visible thoracic excursions, spontaneous movements of the eye lids, and a speaker in the mannequin’s head that broadcasts the voice of a remote operator, allowing for verbal interaction with the participants. For the present trial, the operator was an experienced physician (G.B., K.T., or S.M.)

For the study scenario, participants were given the role of a resident-on-call in the emergency department and were instructed by a senior physician (confederate) to triage a newly arrived patient. Upon entering the simulator room, the participants encountered the patient (simulator) lying on a bed and dressed in a hospital gown. Participants were assisted by a confederate nurse, which was instructed to display a helpful manner, to act only on command, but not to interfere in any way with the assessment and history taking.

### 2.4. Randomization and Intervention

All participants performed the scenario alone and faced a patient with a simulated acute myocardial infarction. In cohort 1, the medical students were randomized (computer-generated numbers) 1:1:1 to three versions of the scenario. The three versions differed only in the reply of the patient to the initial question about the reason for his visit. The patient stated that he had phoned his family physician because of acute chest pain and dyspnoea. The family physician had recommended urgent visit of the emergency department because he judged the situation as (1) medical emergency (control group with no a priori diagnosis); (2) acute myocardial infarction (correct a priori diagnosis group); or, (3) acute pulmonary embolism (wrong a priori diagnosis group). Apart from this randomly allocated initial information, the scenario was identical in all groups: until the end of the scenario, the patient only spoke when asked a question and all patient’s answers were given according to a pre-scripted answer check-list (Table 1).

In cohort 2, physicians were randomized (computer-generated numbers) 1:1 to the control group (no a priori diagnosis) and the wrong a priori diagnosis group.

All participants of the groups with no a priori diagnosis and correct a priori diagnosis were given 4 min assessment time. Participants of the group with the wrong a priori diagnosis were given one additional minute (i.e., 5 min assessment time) to compensate for probing the given diagnosis. At the end of the study time, the senior physician entered the room and announced that he required the participant to temporarily leave his/her patient to help with another more urgent case. While still in the room, the participants were then asked on their presumptive diagnosis and which measures, if any, the nurse should take during their temporary absence during the next couple of minutes.

### 2.5. Data Analysis

Data analysis was performed using video recordings obtained during simulations. Video recordings were analysed independently by two trained raters, who noted all questions asked and all measures taken by the participant. Inter-observer differences were solved by jointly re-watching the video recordings.

### 2.6. Statistics

The primary endpoint was a correct final presumptive diagnosis of an acute myocardial infarction or acute coronary syndrome when asked at the end of the scenario by the senior physician or when previously revealed to the patient and/or the nurse. Mentioning the correct diagnosis as possible differential diagnosis was rated as correct diagnosis. A co-primary endpoint was the composite endpoint of a correct final presumptive diagnosis OR ordering a 12-lead ECG, the rationale being that a timely 12-lead ECG will most likely prevent harm to a patient not correctly triaged as suffering from acute myocardial infarction.

Secondary endpoints were the number and kind of questions asked during history taking; probing of the a priori diagnosis, defined as asking for symptoms or performing examinations specific for the given a priori diagnosis; measures taken during assessment; and the results of the questionnaire. Results of the knowledge part of the questionnaire are reported as percentages of the attainable maximum.

A 20% difference in the primary outcome was considered to be of medical relevance. A power analysis revealed that for the cohort of medical students, approximately 150 participants (50 per group) and for the cohort of physicians approximately 80 (40 per group) had to be included to detect this difference with a power of 0.9 at the 0.05 significance level. A single interim analysis was scheduled after recruitment of half the planned cohort of physicians with two-sided hypothesis testing with boundaries allowing for early stopping either in favour of the null or of the alternative [20,21].

Statistical analysis was performed by SPSS (version 25; IBM, Armonk, NY, USA). Non-parametric ANOVA, Mann–Whitney test, Fisher’s exact test, Univariate and Multivariate regression analysis of variables associated with diagnostic accuracy, and Cohen’s kappa were used as appropriate. A *p* < 0.05 was considered to represent statistical significance. Data are reported as median [Interquartile range {IQR}] unless otherwise stated.

## 3. Results

### 3.1. Medical Students

Out of 316 students offered the possibility, 156 (49%) participated in the voluntary workshops, were included in the trial, and completed the trial as intended. Fifty-two participants (31 females) were randomised to the control group, 52 (34 females) to the correct a priori diagnosis group, and 52 (30 females) to the wrong a priori diagnosis group. No protocol violations occurred and video-recordings of all participants were of sufficient quality for analysis. Complete questionnaire data were available from all participants. Thus, data of all 156 participants were analysed and reported. Groups were well balanced for knowledge and personality (Table 2). Cohen’s kappa for inter-rater reliability was 0.96.

**Figure 1:**
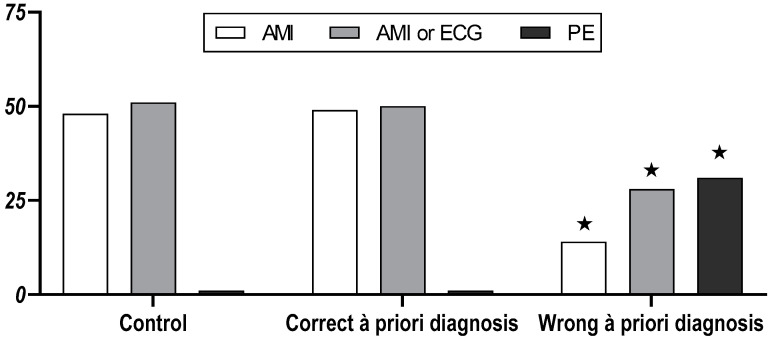
Final presumptive diagnosis of medical student participants after a focussed assessment of a patient with AMI in the control group (*n* = 52), the correct a priori diagnosis group (*n* = 52), and the wrong a priori diagnosis group (*n* = 52). AMI = diagnosis acute myocardial infarction; AMI or ECG = diagnosis acute myocardial infarction OR ordering of a 12-lead ECG; PE = pulmonary embolism. ★ = *p* < 0.05 vs. both other groups.

Primary endpoint: The correct final diagnosis was made by 48/52 (92%) participants in the control group, by 49/52 (94%) participants in the group with the correct a priori diagnosis, and by 14/52 (27%) participants in the group with the wrong a priori diagnosis (*p* < 0.001 vs. both other groups). In the group with the wrong a priori diagnosis, 31/52 (60%; *p* < 0.001 vs. both other groups) indicated pulmonary embolism, i.e., the initially given wrong diagnosis, as their final diagnosis compared to 1/51 (2%) in the control group and 1/51 (2%) in the correct a priori diagnosis group (Figure 1). The composite primary endpoint of a correct final diagnosis or ordering a 12-lead ECG was reached by 51/52 (98%) in the control group, by 50/52 (96%) in the group with the correct a priori diagnosis, and by 28/52 (54%) in the group with wrong a priori diagnosis (*p* < 0.001 vs. both other groups).

Secondary endpoints: Despite having 20% more time available, we observed a similar number of questions asked during history taking in the wrong a priori diagnosis group and the control group (7 [IQR 5–10] vs. 7 [IQR 5–10]; *p* = 0.97). The number of questions asked in the correct a priori diagnosis group 5 [IQR 3–9] was significantly lower than in both the control group (*p* = 0.02) and in the wrong diagnosis group (*p* = 0.032). The number of questions asked per minute of assessment time was significantly higher in the control group (1.8 [IQR 1.3–2.4]) than in both the groups with correct (1.3 [IQR 0.8–2.1]; *p* = 0.02) and wrong (1.4 [IQR 1.0–1.9]; *p* = 0.03) a priori diagnoses. Diagnostic or therapeutic measures taken during the triage scenario are displayed in Table 3. Note that measures taken concurred with the presumptive diagnosis.

Comparisons within the wrong a priori diagnosis group: Participants with a correct and wrong final diagnosis did not differ in gender, knowledge, self-esteem, or personality traits (Table 4).

Moreover, there was no difference in the overall number of questions asked during history taking between participants of the wrong a priori diagnosis group making the correct or false final diagnosis (7 [IQR 4–10] vs. 7 [IQR 6–10]; *p* = 0.42). There was, however, a significant difference in the number of questions asked in the thematic bloc relating to the exploration of chest pain (2 [IQR 1–5] vs. 4 [IQR 3–5]; *p* = 0.016), but not in questions asked in any of the other thematic blocs. Univariate analysis revealed a significant correlation of making the correct presumptive diagnosis with asking of the quality of chest pain (beta 0.47; *p* < 0.0001), the radiation of chest pain (beta 0.47; *p* < 0.0001), and having had similar symptoms before (beta 0.28; *p* < 0.045). In addition, there was a non-significant trend for a correlation with asking of the localization of chest pain (beta 0.26; *p* < 0.066) while none of the other questions reached a *p* value of < 0.10. In multivariate analysis, asking about both the quality of chest pain (beta 0.30; *p* = 0.049) and radiation of chest pain (beta 0.30; *p* = 0.049) emerged as independent predictors of making a correct presumptive diagnosis. Interestingly, the knowledge that the quality of chest pain and radiation are typical symptoms of an AMI, as evidenced by the questionnaire, was not significantly correlated with the likelihood of asking the corresponding questions during history taking (correlation coefficients −0.01 and 0.09, *p* values 0.96 and 0.54 respectively).

Probing: In the wrong diagnosis group, we observed only limited probing of the a priori diagnosis, defined as asking for specific symptoms or risk factors of pulmonary embolism, with no significant difference between participants making the correct or wrong final diagnosis (1 [IQR 1–2] vs. 1 [IQR 0–1]; *p* = 0.20).

Likewise, compared to the control group, we observed in the correct a priori diagnosis group significantly fewer probing questions, i.e., questions relating to chest pain symptoms (3 [IQR 2–4] vs. 4 [IQR 3–5]; *p* = 0.040) and risk factors of coronary disease (0 [IQR 0–2] vs. 1 [IQR 0–2]; *p* = 0.037), while there was no difference between these groups in the number of questions in any of the other thematic blocs.

### 3.2. Physicians

The trial was terminated after the interim analysis since the boundary for efficiency, i.e., rejection of the null hypothesis, had been crossed for the primary endpoint (correct diagnosis) and the boundary for futility, i.e., in favour of the null hypothesis, had been crossed for the co-primary endpoint (correct diagnosis or 12-lead ECG). At the time of the interim analysis, 44 (24 female) physicians had been included and randomised to the control group (*n* = 21; 10 female) and to the wrong a priori diagnosis group (*n* = 23; 14 female). All physician participants completed the scenario as intended, no protocol violations occurred, all video-recordings were of sufficient quality for analysis so that data of all 44 participants were analysed and reported. Groups were balanced for personality and years of professional activity after graduation from medical school (10 [IQR 7–16] vs. 8 [IQR 5–17] years; *p* = 0.21). Cohen’s kappa for inter-rater reliability was 0.94.

**Figure 2:**
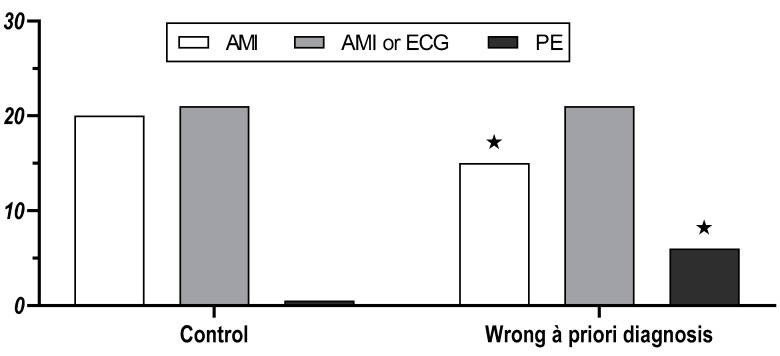
Final presumptive diagnosis of the physician participants after a focussed assessment of a patient with AMI in the control group (*n* = 21), and the wrong a priori diagnosis group (*n* = 23). AMI = diagnosis acute myocardial infarction; AMI or ECG = diagnosis acute myocardial infarction OR ordering of a 12-lead ECG; PE = pulmonary embolism. ★ = *p* < 0.05 vs. both other groups.

Primary endpoint (Figure 2): A correct final diagnosis was reached by 20/21 (95%) physicians in the control group and 15/23 (65%) in the wrong a priori diagnosis group (*p* = 0.023). In the wrong a priori diagnosis group, 6/23 (26%) indicated pulmonary embolism, i.e., the initially given wrong a priori diagnosis, as their final diagnosis, while 2/23 (9%) indicated another diagnosis. The composite primary endpoint of a correct diagnosis or ordering a 12-lead ECG was reached by 21/21 (100%) in the control group and by 21/23 (91%) in the wrong diagnosis group (*p* = 0.49).

Secondary endpoints: Participants with a correct and final diagnosis did not differ from those with a wrong final diagnosis with regard to gender (*p* = 0.57), personality, and years of professional activity after graduation from medical school (*p* = 0.15). A similar number of questions were asked during history taking in the control group, in the wrong a priori diagnosis group (10 [IQR 8–12] vs. 11 [IQR 8–12]; *p* = 0.64), and from participants with a correct and a wrong final diagnosis in the wrong a priori diagnosis group (11 [IQR 9–13] vs. 10 [IQR 8–12]; *p* = 0.27).

In the wrong a priori diagnosis group, only radiation of chest pain (beta 0.65; *p* < 0.0001) reached a *p* value of <0.10 for association with the correct presumptive diagnosis.

Participants were asked three [IQR 1–4] probing questions in the wrong a priori diagnosis group with no significant difference observed between correct or wrong final diagnoses. In the wrong a priori diagnosis group, participants making the wrong final diagnosis did not differ in self-esteem or personality traits from participants making the correct final diagnosis.

Participants took six [IQR 5–7] diagnostic or therapeutic measures with no significant differences between the groups. Like in the student cohort, measures taken by the physicians concurred with their presumptive diagnosis: Participants of the wrong a priori diagnosis group more often monitored oxygen saturation with pulse oximetry (13/23 (57%) vs. 5/21 (24%); *p* = 0.028), measured d-Dimers (12/23 (52%) vs. 3/18 (17%); *p* = 0.009), and examined the patient’s legs (8/23 (35%) vs. 1/20 (5%); *p* = 0.016), while participants of the control group administered nitro-glycerine (16/21 (76%) vs. 4/19 (21%); *p* < 0.0001) more often.

## 4. Discussion

In this prospective randomized trial, a wrong a priori diagnosis resulted in a substantial likelihood of a wrong final diagnosis. In addition, a wrong a priori diagnosis was associated with a different pattern of measures taken during the patient’s encounter, including both omissions and commissions. The presence of an a priori diagnosis, regardless whether correct or false, resulted in less questions asked during history taking and probing of a priori diagnoses occurred rarely.

Diagnostic errors can be defined as a diagnosis that is missed, wrong, or delayed, as detected by some subsequent definitive test of finding [22,23]. Most current knowledge on diagnostic errors is based on retrospective and observational studies that are prone to hindsight bias [11]. So far, there are only few prospective data on diagnostic errors and, to the best of our knowledge, this is the first prospective randomized controlled trial on this topic.

In a real-life study, one serious medical error per six to eight patient-days was observed in pediatric intensive care with residents involved in approximately half of the errors [14]. A diagnostic-scenario based prospective study in physicians reported a high rate of incorrect diagnoses (43–56%) [24]. Diagnostic accuracy was positively correlated with the number of critical cue requests and was not affected by the physicians’ experience. Of note, approximately 90% of incorrect diagnoses were followed by inappropriate management [24]. A case-vignette-based prospective study in medical students demonstrated a high rate (43%) of diagnostic errors, the main causes of diagnostic errors being inadequate diagnostic skills, inadequate knowledge, and faulty context generation, while premature closure was noted in 10% [12]. By contrast, the present study demonstrates that knowledge and diagnostic skills did not differ between students making the correct or wrong diagnosis. When faced with unannounced, standardized patients with uncomplicated ambulatory complaints, physicians provided error-free care 73% of the time [15]. However, that percentage decreased to 9% with the addition of bio-medically and contextually complicating factors that required an alternative plan of care. In keeping with our results, diagnostic errors occurred because physicians did not probe in response to contextual red flags [15]. Expert patients’ record review followed by a discussion with the treating physician revealed an error rate of 14% and patient harm in 11% of the cases; the most common causes being mistakes, meaning that treating physicians did not realize their actions were incorrect [6].

Diagnostic errors may lead to harm by omission (e.g., delayed or no treatment of the not diagnosed condition) and/or commission (e.g., treatment of the wrongly diagnosed condition) [7]. In the present trial, the wrong a priori diagnosis influenced the measures taken during the triage scenario concurrently to the presumed diagnosis. With the exception of ordering a 12-lead ECG, the pattern of omissions and commissions was very similar in both medical students and physicians. A delayed or initially missed diagnosis in acute myocardial infarction leads to harm since delays in reperfusion therapy results in increased morbidity and mortality [25,26].

History taking and assessment are seen as the most sensitive parts of the diagnostic process [6,27,28,29,30]. This process, known as diagnostic reasoning, is a judgement process that takes place under uncertainty and includes considerations of possible diagnoses, harms, and benefits of diagnostic testing, and the patient’s preferences and values. So far, diagnostic reasoning is understood as a dual process [31]. It is divided in an analytical deliberate rational process guided by critical thinking and in a non-analytical unconscious, intuitive, and automatic process [27,31,32].

The approach known as pattern recognition, which is a part of the non-analytical system, is a frequently used procedure in the diagnostic process [27,29,33]. Though pattern recognition is a fast and successful method, it can lead to errors due to premature closure [27].

Successful preventive strategies against diagnostic errors require the understanding of underlying mechanisms. There are, however, different kinds of diagnostic errors with different underlying mechanisms [34,35,36,37,38,39]. The present trial focussed on diagnostic errors induced by or correlated with an a priori diagnosis. Our results are in keeping with the notion that diagnostic errors result less from a lack of medical knowledge than on a misinterpretation of medical facts [40,41,42]. We observed insufficient probing of a priori diagnosis regardless of whether it was correct or wrong. In addition, differential diagnoses were not systematically assessed regardless of whether there was a correct or wrong a priori diagnosis. Moreover, very few general and unspecific symptoms (e.g., chest pain, dyspnoea) may be perceived as confirmatory proof of an a priori diagnosis. Taken together, an a priori diagnosis is able to induce a fixation error. This fixation error encompasses aspects of a variety of cognitive biases like premature closure, confirmation bias, anchoring bias, and framing effects [38,39]. Asking questions on specific characteristics of chest pain (i.e., quality, radiation) was associated with a high likelihood of making the correct diagnosis. This may be explained by the hypothesis that hearing these typical symptoms from the patient did trigger a pattern recognition process. Surprisingly, the knowledge that the quality of chest pain and the radiation of chest pain are typical symptoms of a myocardial infarction did not translate into asking the corresponding questions during history taking.

Participants in the wrong a priori diagnosis group making the correct or wrong final diagnosis did not differ regarding gender, experience, knowledge, personality traits, quantity of patient’s history taking, and several qualitative aspects of history taking like probing. Thus, apparently similar personalities with similar knowledge followed a similar pattern of assessment and were merely mislead or rewarded by the contextual factors like the correctness of their a priori diagnosis or asking few specific questions.

Our findings have several implications: First, as a priori diagnoses are frequent in patients’ encounters, practitioners should be aware that in the presence of an a priori diagnosis, diagnostic errors and especially fixation errors may occur easily. Second, preventive measures against diagnostic errors in the presence of an a priori diagnosis are sufficient probing and systematic history taking, which includes asking key questions. This should be emphasized in medical education for both students and physicians. Third, simulation is a suitable tool to expose diagnostic errors and teach and train preventive measures. Fourth, the present trial demonstrates that prospective randomized controlled trials on diagnostic errors are feasible and especially so in the settings of simulation.

Limitations are the single-centre design and the limitations of simulator-based studies, which mainly include the absence of real patients and real environment. However, simulation allowed highly standardised conditions for a large number of participants, which would be very difficult to achieve in real life. Moreover, exposing real patients deliberately to a wrong a priori diagnosis is ethically not justifiable. The inclusion of medical students may be regarded as further limitation. However, the high success rates of the control group, i.e., the group without an a priori diagnosis, demonstrates that our participants, being medical student or physician, had the knowledge and skills to master our triage scenario. The present trial assessed one condition and one wrong a priori diagnosis, and the results can therefore not necessarily be generalized to constellations of other diseases. However, a particular strength of this study is the selection of a scenario (myocardial infarction) and a wrong presumptive diagnosis (pulmonary embolism) that are commonly associated with diagnostic errors in real cases [43].

## 5. Conclusions

The present trial demonstrates that a wrong a priori diagnosis increases the likelihood of a wrong final diagnosis and significantly influences history taking and patient management.

## Figures and Tables

**Table 1:** Checklist of the patient’s answers to questions asked by the participants during history taking.

Questions of the Participants	Answers of the Patient
**Exploration of chest pain (chest pain bloc)**	
Localisation	In the middle of the chest behind the breastbone
Quality	Pressing and constricting
Radiation	Yes, to the left elbow and neck
Severity	Severe. When a pain score is asked: 8 out of 10
Aggravating or relieving factors	None
Previous experience	Never experienced something similar before
Duration	Continuous pain over the last 30 min
Circumstances	Sudden onset while sitting at the desk working. When asked: no preceding physical activity or unusual stress
**Associated symptoms**	
Nausea or vomiting	Slight Nausea, no vomiting
Sweating	Initially heavy sweating, that now has ceased
Fear	Initially strong fear of death, now frightened
Dyspnoea	Slight shortness of breath
**Cardiovascular risk factors**	
Smoking	One package cigarettes a day for the last 30 years
Hypertension	No. Blood pressure normal, according to GP
Diabetes	No
Blood fats	Normal, according to GP
Family history	Mother died due to a stroke with 70 years; father 82 years old with dementia; two healthy brothers
**Previous illness and medication**	
Prior diseases	None
Prior hospitalisations	Appendectomy when 16 years old
Current medication	None
Allergies	Not known
Last visit with GP	Normal “check-up” one year ago
**Social history**	
Age	49 years old
Next of kin	Married, no children
Profession/occupation	Accountant in banking
Recreation activities/hobbies	Cycling, fitness centre, traveling
**Exploration for pulmonary embolism**	
Cough	None
Haemoptoe	No
Chest pain aggravated by breathing	No relation of chest pain with breathing activity
Painful or swollen leg(s)	No
Previous thrombotic event	No
Immobilisation	No. When specifically asked: no trauma, no operation, no recent travel
Risk factors for thrombotic event	When specifically asked: patient not aware of cancer, coagulopathy, coagulopathy in family

**Table 2:** Questionnaire data of the student participants.

	Control Group (*n* = 52)	Correct a Priori Diagnosis Group (*n* = 52)	Wrong a Priori Diagnosis Group (*n* = 52)
Female:male	31:21	34:18	30:22
AMI knowledge %	50 [33–67]	50 [33–67]	50 [50–67]
Emergencies knowledge %	48 [37–60]	42 [27–58]	44 [37–54]
ECG knowledge %	42 [25–54]	33 [29–50]	42 [25–58]
Self-esteem (Rosenberg)	23 [20–26]	24 [20–26]	22 [17–24]
Neuroticism	2.8 [2.2–3.3]	2.9 [2.2–3.3]	2.9 [2.5–3.5]
Extraversion	4.3 [3.7–4.8]	4.2 [3.8–4.8]	4.2 [3.7–4.7]
Openness	4.0 [3.7–4.6]	4.5 [3.9–4.8]	4.2 [3.8–4.6]
Agreeableness	4.7 [4.3–5.0]	4.7 [4.5–5.0]	4.7 [4.3–5.0]
Conscientiousness	4.7 [3.8–4.8]	4.5 [3.9–4.8]	4.3 [3.8–5.0]

Data are medians [Interquartile range {IQR}]; AMI = acute myocardial infarction. ECG = Electrocardiogram. Data on knowledge are expressed as percentages of the maximum attainable result. Personality traits (neuroticism, extraversion, openness, agreeableness, and conscientiousness) are based on 6 points Likert scales. There are no significant differences between the groups.

**Table 3:** Measures taken by the student participants.

	Control Group (*n* = 52)	Correct a Priori Diagnosis Group (*n* = 52)	Wrong a Priori Diagnosis Group (*n* = 52)
Number of measures taken (*n*)	4.5 [3.0–6.0]	5.0 [3.0–5.5]	5.0 [4.0–5.0]
Supplemental oxygen (*n*; %)	28/52 (54%)	29/52 (56%)	16/52 (31%) *^¶^
Cue ECG monitoring (*n*; %)	25/52 (48%)	30/52 (58%)	28/52 (54%)
Blood pressure and heart rate measured (*n*, %)	43/52 (83%)	39/52 (75%)	35/52 (67%)
Oxygen saturation measured (*n*; %)	3/52 (6%)	1/52 (2%)	3/52 (6%)
Chest auscultation (*n*; %)	32/52 (62%)	25/52 (48%)	42/52 (81%) *^¶^
Examination of legs (*n*; %)	0/52 (0%)	0/52 (0%)	9/52 (17%) *^¶^
Morphium given (*n*; %)	32/52 (62%)	29/52 (56%)	12/52 (23%) *^¶^
Nitro-glycerin given (*n*; %)	25/52 (48%)	22/52 (42%)	4/52 (8%) *^¶^
Aspirin given (*n*; %)	9/52 (17%)	13/52 (25%)	1/52 (2%) *^¶^
Heparin given (*n*; %)	0/52 (0%)	4/52 (8%)	3/52 (6%)
Chest Radiograph ordered (*n*; %)	0/52 (0%)	0/52 (0%)	9/52 (17%) *^¶^
Chest CT ordered (*n*; %)	0/52 (0%)	0/52 (0%)	9/52 (17%) *^¶^
Blood sample taken (*n*; %)	24/52 (46%)	25/52 (48%)	43/52 (83%) *^¶^
Troponin requested (*n*; %)	14/52 (27%)	16/52 (31%)	4/52 (8%) *^¶^
D-Dimer requested (*n*; %)	0/52 (0%)	3/52 (6%)	22/52 (42%) *^¶^

Data in the first row are medians [Interquartile range {IQR}]; Digits in the following rows indicate the number of participants of the given group taking the specified measure. * = *p* < 0.05 vs. control group; ¶ = *p* < 0.05 vs. correct diagnosis group.

**Table 4:** Comparisons of student participants randomized to the wrong diagnosis group.

	Final Diagnosis Acute Coronary Syndrome (*n* = 21)	Final Diagnosis Pulmonary Embolism (*n* = 31)
Female:male (*n*)	13:8	17:14
AMI knowledge %	67 [50–67]	50 [50–67]
Emergencies knowledge %	45 [30–53]	42 [39–57]
ECG knowledge %	33 [25–58]	42 [33–58]
Self-esteem (Rosenberg)	22 [20–23]	22 [16–24]
Neuroticism	2.8 [2.7–3.2]	3.0 [2.5–3.8]
Extraversion	4.3 [3.8–4.7]	4.2 [3.7–4.5]
Openness	4.3 [3.8–4.7]	4.0 [3.7–4.5]
Agreeableness	4.7 [4.3–5.0]	4.7 [4.3–5.0]
Conscientiousness	4.5 [4.0–5.0]	4.2 [3.7–5.0]
Number of measures taken (*n*; %)	5.0 [4.0–5.0]	5.0 [4.0–6.0]

Data are medians [Interquartile range {IQR}]; AMI = acute myocardial infarction. Data on knowledge are expressed as percentages of the maximum attainable result. Personality traits (neuroticism, extraversion, openness, agreeableness, and conscientiousness) are based on 6 points Likert scales. There are no significant differences between the groups. There are no significant differences between the two groups.

## Data Availability

The data (i.e., video-recordings) are not publicly available due to strict confidentiality guaranteed to participants.
